# Continuous deep sedation and homicide: an unsolved problem in law and professional morality

**DOI:** 10.1007/s11019-015-9680-3

**Published:** 2015-12-29

**Authors:** Govert den Hartogh

**Affiliations:** Department of Philosophy, University of Amsterdam, Staten Bolwerk 16, 2011 ML Haarlem, The Netherlands

**Keywords:** Continuous deep sedation, Double effect, Medical exception, Palliative sedation, Slow euthanasia, Withholding artificial hydration

## Abstract

When a severely suffering dying patient is deeply sedated, and this sedated condition is meant to continue until his death, the doctor involved often decides to abstain from artificially administering fluids. For this dual procedure almost all guidelines require that the patient should not have a life expectancy beyond a stipulated maximum of days (4–14). The reason obviously is that in case of a longer life-expectancy the patient may die from dehydration rather than from his lethal illness. But no guideline tells us how we should describe the dual procedure in case of a longer life-expectancy. Many arguments have been advanced why we should not consider it to be a form of homicide, that is, ending the life of the patient (with or without his request). I argue that none of these arguments, taken separately or jointly, is persuasive. When a commission, even one that is not itself life-shortening, foreseeably renders a person unable to undo the life-shortening effects of another, simultaneous omission, the commission and the omission together should be acknowledged to kill her. I discuss the legal and ethical implications of this conclusion.

## The question

Of the various forms of ‘palliative sedation’, as it is usually called by now,^1^ the procedure that raises most ethical and legal concerns is continuous deep sedation: sedation to unconsciousness that is meant to go on until the patient dies.^2^ All guidelines and position statements that have been published in recent years by professional organisations and consensus groups permit this only as a last step in a process of titration that in its earlier stages has failed to relieve the patient’s refractory symptoms. In practice patients who do not want to wait and see whether the first steps in that process are really effective are sometimes brought into a state of coma directly and kept in it until they die.^3^

The dosage of sedatives normally needed to make a person fully unconscious is less than half the dosage standardly used in anesthetic procedures, and obviously even that last dosage would not be used if any life-shortening effects could be expected. Hence by itself deeply sedating a patient in the medically appropriate way probably does not shorten his life.^4^ This is stressed by most guidelines. If a minimal risk remains, as a few guidelines suggest,^5^ this need not be a concern for the doctor’s conscience, let alone for the criminal law: many of our daily activities involve such minimal risks.^6^

But when a patient’s consciousness is lowered, even moderately, that is to a level short of full unconsciousness, he will no longer be able to eat or drink. Hence a decision has to be made whether or not to start (or, occasionally, to continue) artificial nutrition and, in particular, hydration. In some cases the patient will already have died before it becomes necessary to answer that question, but in other cases the question will eventually require an answer. In the case of deep sedation it will in some countries be decided almost routinely to abstain from the artificial provision of fluids, in other countries they will be provided in most cases.^7^ If it is decided to withhold fluids and the patient goes on to live for a week or longer, the real possibility exists that he eventually dies from dehydration. Even if this outcome may be hastened by the patient’s illness and general weakening, and the illness can therefore be viewed as co-determining the exact moment at which the patient dies, the dehydration is itself a co-determinant of the moment of death as well.^8^ Because we are talking about patients in the final stage of a fatal illness, it is probable that in some cases dehydration may have this life-shortening effect already after a few days. Should we in these cases think of the doctor as ending the life of the patient?^9^

If we consult the existing guidelines we will, remarkably, find no anwer to that question. That does not mean that they are not concerned about the issue. Most guidelines state as a condition for starting deep and continuous sedation that a patient has a reduced life expectancy, sometimes specified as ‘hours or days’,^10^ sometimes as ‘days or weeks’.^11^ If a more specific upper limit is given, it is usually put at 14 days.^12^ Because that is about the maximum time that people can live without fluids, one may guess that the reason for this requirement has something to do with a possible life-shortening effect, and this is confirmed in the few cases in which a reason is given.^13^ So the guidelines are certainly concerned about the *dual procedure*, as I will call it, of deep and continuous sedation and withholding fluids, when the patient has a life expectancy beyond a certain number of days (4–14), and that concern seems to be motivated by the possibly life-shortening effect of that procedure. Nevertheless, the guidelines do not characterize this dual procedure, when it causes the death of the patient, as a form of killing.

Should they? From the beginning of the ethical and legal discussion about ‘terminal sedation’ (as it was then called) the suspicion has been voiced that this medical procedure sometimes amounts to ‘slow euthanasia’.^14^ It has been a primary concern of the guidelines to refute that accusation. That is why they stress that even deep continuous sedation, if done by using appropriate dosages of sedatives, by itself has no life-shortening effects. Such effects could only occur when that decision is followed by the decision not to administer artificial nutrition and hydration. And for this dual procedure they have addressed the concern by setting upper limits to the life-expectancy of the patient to be sedated.

Has this strategy been successful? I will discuss that question in § 4, concentrating on the different specifications of the upper limit (roughly between 4 and 14 days) provided by the guidelines. I will argue that if the upper limit is meant to prevent the dual procedure from probably amounting to a form of homicide, it should be put at a lower level than most guidelines propose. But even if we accept an upper limit of 14 days, the question remains how we should describe the dual procedure, morally and legally, when the life expectancy of the patient exceeds this limit. Shouldn’t we squarely face the fact that it is probably a form of homicide, and be consistent in drawing the legal and moral consequences of that insight? In § 2 I will argue that we should. In § 3 I will discuss the legal meaning of this conclusion, and consider in particular whether the doctor who has committed this ‘offence’ -as it will be considered in all jurisdictions, at least *prima facie*- has any legal defenses. Finally, in § 5 I will first ask how the guidelines could be updated to take my conclusions into account. I will then go on to argue that if we do not want to update them because of the price we have to pay for such updating, we are committed to recognize the legitimacy of at least one form of physician-assisted death.

As this outline of my argument shows, it is not my aim in this paper to arrive at a final moral evaluation of the cases in which the dual procedure, on my view, amounts to a kind of homicide. There could still be considerations that can be appealed to in order to justify them, or some of them, as many people believe that other forms of physician-procured or -assisted death can be justified. Conversely, I certainly do not want to argue in favour of a policy of always providing fluids.

What do I mean by ‘homicide’? One can kill someone by accident, but homicide refers to the non-accidental causing of the death of another human being. This can still be done negligently or recklessly. But if a person has a life-expectancy of more than 2 weeks, it is to be expected that she will die from dehydration if she doesn’t eat or drink. A doctor who deeply sedates her and doesn’t provide her with artificial nourishment and hydration should be considered to know this. Hence his action is neither negligent nor reckless.

So we are talking about a kind of homicide that is characterized by foresight. In addition to this cognitive element a volitional element is involved in homicide: that you clearly foresaw the death of the other person didn’t motivate you to abstain from the action. In that sense you can be said to have endorsed the result. However, you do not need to have intended it. As we will see (§ 3), in some legal systems and according to some moral views intention is relevant for the classification and evaluation of a homicide. But it is not required for considering it a homicide to begin with. To quote the classical case, if you arrange for a ship or an airplane to be blown up by dynamite in order to pocket the insurance money, you will not intend the death of the crew, but only endorse it as a, possibly unwelcome, side-effect of executing your plan. But that you will commit a homicide is beyond dispute.

To characterize an action as an act of killing or a homicide is not by itself to condemn it, for it is possible for a homicide to be, all things considered, morally and legally justifiable. ‘A permissible killing’ is not an oxymoron. On the other hand, the classification is always relevant for the moral and legal evaluation of the action. The action is at least in need of a justification.

My argument will be fairly complex but the basic point is a simple one. Deep sedation until death is a way to prevent extreme suffering at the end of life that is seen by many as an alternative to all forms of physician-assisted death. It often is such an alternative, though even in those cases it is an open question which alternative is to be preferred. But in other cases the patient’s life could predictably be extended by providing artificial nutrition and hydration. If in those cases it is decided not to provide these, for whatever reason, this dual procedure is not an alternative to all forms of physician-assisted death. It *is* a form of physician-assisted death.

## ‘Slow euthanasia’?

When is it unproblematic to start continuous deep sedation without administering any fluids? The first observation that should be made in finding an answer to this question is that in each dying process there comes a point at which the patient spontaneously stops eating and drinking because he loses the craving for it. That is a normal part of dying that should always be respected. To start artificial hydration in such a case is an unnecessary prolonging of the dying process, and may induce additional forms of suffering, for example by causing or increasing oedema, ascites or bronchial secretions.^15^ Hence it may not only be futile but even cruel treatment. If sedation begins when this point has already been reached, it is obvious that artificial hydration should not even be considered, even when we assume that, as a result of deep sedation, it will no longer cause suffering.

Some guidelines suggest that in almost all cases in which continuous deep sedation is decided upon, the patient has already spontaneously stopped taking fluids.^16^ That contention is open to doubt,^17^ but even if it is true, the few cases left are worth discussing. For even if we may assume that in these cases the patient’s body will soon start resisting any further intake, as long as that point has not been reached it is an open question whether or not the dual procedure may shorten his life. And a killing is a killing, even if it occurs only a few minutes before the moment at which the person would have died from ‘natural’ causes.

It is a constantly recurring theme in the guidelines that the decision to start or withhold the artificial provision of food and fluids has to be made on independent grounds. Neither decision should be automatically implied in the decision to start sedation.^18^ This makes sense as long as the patient has not become fully unconscious. Even at moderate levels of unconsciousness she may still experience feelings of hunger and thirst, or suffer from a dry mouth on the one hand, whereas providing food and drink may cause its own forms of suffering (for example resulting from obstipation) on the other.^19^ In such cases it is true that these effects should be balanced against each other. But as soon as the patient is in a state of coma, either as the final outcome of titration or as the result of a non-titrating policy, such balancing makes no sense anymore. For the patient, we assume, does not experience anything at all and hence does not suffer. The only reason we could then have for providing fluids is that death from dehydration is thereby prevented.^20^ If that reason is not a valid one, we should always abstain from providing food and fluids, once we have decided to start deep sedation until death. No further balancing of reasons is needed.

The Dutch and Flemish guidelines and a few others take the view that in such cases artificial hydration should, indeed, always be withheld or withdrawn because it is to be considered futile.^21^ It is futile because for a patient who has permanently, if not irreversibly, lost consciousness, it doesn’t matter in which condition she is, not even if she fully dries out.^22^ But how can a treatment be futile that prolongs her life, or at least prevents it from being shortened? In that judgment it is presupposed that merely being alive has no value of its own for the living person, if that life isn’t the vehicle of other goods, a conscious and not too painful experience of the world to begin with.^23^ On this assumption mere biological life cannot be good *for you*, if you do not have and never again will have a point of view from which you can endorse (or, for that matter, deny) that evaluation.

Personally I share this view, but it should be observed that even in the secularized societies of the Netherlands and Flandres it is not shared by everyone. According to an alternative view, mostly held on religious grounds, mere biological life is by itself a basic human good,^24^ and therefore always to be protected, at least by ‘ordinary means’. And artificial hydration is not an ‘extraordinary means’, if it prolongs life and doesn’t cause any suffering.^25^ In a pluralist society this is a view that should be respected. Patients may therefore legitimately request artificial hydration, in some religious communities (and in less seculartzed societies) it may legitimately be the *default*, and in some religious hospitals the standard.

If this alternative view is correct, there might be a direct argument to the conclusion that failing to provide artificial nutrition and hydration to a sedated patient amounts to killing her, when she has a life-expectancy beyond the maximum. Consider a doctor who fails to give antibiotics to a patient with pneumonia who is otherwise perfectly healthy, and thereby causes the patient to die. Even though this is failing to act rather than acting, it is commonly seen as homicide, because the doctor has a duty of care to his patient. On this view causing death by failing to provide a non-futile treatment has to be considered homicidal.

I will not try to assess this argument, for what I want to focus on, is a different set of arguments. That artificially administering fluids is a futile procedure, when its only aim can be to prolong mere biological life, is an equally respectable view.^26^ This view, anyway, is the starting-point of the most plausible, and hence for me the most challenging argument that can be given to support the conclusion that the dual procedure never amounts to killing, even if it results in an earlier death. That is the argument that I will scrutinize in the remainder of this section.

The argument proceeds as follows. There is no problem with sedation, even to full unconsciousness, because it has no life-shortening effects by itself. There is no problem with abstaining from artificial hydration either, because it is only a form of allowing to die, and the treatment may properly be considered futile. Neither of these decisions is a form of killing. And so the combination of the two cannot be a form of killing either.^27^

Is this a valid argument? If it is, the guidelines are mistaken in stipulating a maximum life expectancy at all. For even in cases in which we could expect the patient eventually to die from dehydration rather than from his illness, that would not be a problem. Indeed, it would not be a problem to sedate patients, e.g. with MS or Huntington’s disease, without administering to them any fluids, even if they still have a life expectancy of several years.

But the argument is fallacious. Suppose that of two actions, for example both consisting of the injection of a certain amount of muscle relaxants, neither has a lethal effect by itself, but the combination has. Knowingly following a policy consisting of both actions surely amounts to killing. This much is obvious. Actually doing the first action with the intention to do the second is already considered a crime by the law, whether or not the second action will be performed. But even when the first action has been done without this intention, clearly it is impermissible to go on and do the second.

Does it make a difference to the plausibility of the argument that in the sedation case we are talking about the combination of an action and an omission? Consider the following analogy. Someone wants to get rid of his enemy, but in order not to be liable to prosecution for murder, he first sedates his victim to unconsciousness and then sees to it that no food or drink is being provided to him. The victim dies after 2 weeks. Of course the scheme will be unsuccessful, and the criminal convicted of murder, for his two sub plans (to sedate and to avoid the administering of food and drink) only make sense as constitutive elements of one plan: to bring about the death of the victim.

On being confronted with a similar counter-example Torbjörn Tännsjö suggests that even if the policy of our criminal is morally on a par with murder, *strictu senso* it isn’t a form of killing.^28^ If the law considers it to be homicide, it is using a legal fiction. On his view the policy consists of an action of taking away someone’s liberty and then allowing her to die from hunger and thirst. But if he is right, one doesn’t kill a person either by throwing her into the sea from an airplane. That policy also consists of an action: displacing the victim into the sea, and a failure to act: allowing her to drown, and neither the action nor the omission is a killing by itself.

It could be objected that the combination of the two is a killing in this case because the process which, if not interrupted, will end the victim’s life (drowning), is initiated by the killer; it is only when one hasn’t initiated the process which ends her life that one can be said to merely allow her to die. On that view a person who stops eating and drinking does not kill herself either, she only allows herself to die from an independent process, to which we are all subjected, of dying from lack of food and drink. We only don’t notice that natural process because we routinely block it by our actions. That view is controversial itself.^29^ But even if it would be correct, sedating a person is quite another matter, for it means actively *disabling* the person to block that ‘natural’ process herself. If you don’t compensate for that by taking over the blocking, you are thereby killing her. Hence, by sedating a person with a more than minimal life expectancy you are killing that person, unless you artificially provide her with food and drink, just as you would kill her by throwing her into the sea, unless you took active steps to get her out. Such actions are incomplete killings which are completed by ‘allowing nature to take its course’. The action and the abstaining are successive elements of a single policy which has someone’s death as its foreseeable effect.^30^

It is true that in many cases the doctor would have decided to start continuous deep sedation even if in that case he had been obliged to start administering fluids as well. That first decision can therefore truly be seen as independent of the second one, the decision to withhold hydration.^31^ That second decision, however, certainly is not made independently of the first one, and it is this second decision which, therefore, should be seen as part of a single procedure which should be evaluated as a whole. Similarly, if we throw a person into the sea from an airplane for some independent, maybe even legitimate reason, for example to lighten the falling airplane, and only then decide not to drop the lifejacket that she needs to bring herself into safety, we are still killing that person.

A standard reply to this line of reasoning is that artificial nutrition and hydration are medical actions that can only be started with the informed consent of the patient (or his representative), which he is fully authorized to refuse. According to this objection the argument about futility is a red herring; what we should say instead is that continuous deep sedation is justified by the consent, given by the patient to to a medically indicated treatment, and abstaining from artificial hydration by his refusal to have it administered.^32^ This argument seems to have convinced even those authors who most prominently have suggested that the dual procedure should sometimes be described as ‘slow euthanasia’. It is true that if the patient is not prepared to consent to the provision of artificial hydration once sedation has started, it is not an option for the doctor to provide it, whether or not he considers it futile treatment. But this means that in deciding whether or not to start sedation, he has to take into account that he will have to abstain from artificial hydration. When he decides to go on, even though the patient has a life-expectancy of more than 2 weeks, he still knowingly initiates a course of events that will cause the death of the patient and at the same time disables her to prevent that effect from occurring. That analysis still applies, even though the patient doesn’t want to prevent that effect from occurring. Hence the doctor knows that he will probably cause the patient to die by dehydration, and therefore, by following the dual procedure, will probably kill her, albeit it with her consent.^33^ Similarly if you throw someone into the sea with his consent, knowing that he will then refuse to be rescued, you are still causing his death by drowning, albeit on his request. It is still the same ‘salami-slicing technique’^34^ to consider the two decisions independently of each other.^35^

The analogies I have used in this section (incarceration, throwing a person into the sea) might suggest that I argue for a strong normative conclusion. But, as I pointed out already at the end of § 1, that is not the case. By saying that the dual procedure should sometimes be recognized to be a kind of homicide, I do not mean to exclude the possibility that it can be justified. The only normative conclusion that I am prepared to draw at this point is that, in order to avoid moral and legal condemnation, the doctor should be able to offer an adequate justification.

## The legal argument

In § 2 I have argued that a patient who dies of dehydration as a result of the dual procedure, has been killed by his doctor. The doctor can morally and legally be held responsible for this outcome, if it could *ex ante* have been predicted with sufficient probability. What is the legal meaning of this finding? It is difficult to discuss this question on a fully general level because of the bewildering amount of structural divergence between laws of homicide.^36^ Some jurisdictions recognize ending someone’s life on his explicit request as a separate crime, besides murder and manslaughter, others don’t. Conceptions of *mens rea* differ widely: in most European jurisdictions what matters is foresight of death as a causal consequence of one’s action, not whether this consequence is strictly intended, but in common law countries the intention of the defendant may also be relevant, though in very different and not always very clear ways. In some countries the consequence must have been virtually certain for a classification as ‘murder’ to be applicable, in others a substantial probability is sufficient, but even these jurisdictions may disagree about wat counts as a substantial probability.^37^ To differentiate murder from manslaughter (or first and second degree murder) premeditation is sometimes essential, but even if it is, the term has not always the same meaning: in some US states it requires some time spent in deliberation, in others it doesn’t.

Nevertheless, in spite of all this variation, we can be sure that if the patient actually dies as a result of dehydration, and this has been foreseen, it is a case of homicide, however it is to be classified further. If the patient has consented to both elements of the procedure, it will be a case of ending a person’s life on his explicit request in countries in which this is a separate crime (for example: Denmark, Germany, Italy, the Netherlands, Norway). In many other countries or states it will be a case of murder, but in some countries (e.g. Singapore, Australia) the *ex ante* probability of the outcome will not be enough to warrant that description. In those countries the proper classification will be manslaughter (or second degree murder). Finally, in some countries (e.g. France, the UK) a lower degree of probability will be enough for murder, if death as a consequence is not merely foreseen but strictly intended.

It should be recognized, however, that in any concrete case it will be difficult, perhaps impossible, to *prove* that the patient did not die, at the actual moment he did, from his fatal illness alone. And for a conviction virtual certainty is required. For the same reason the risk of prosecution run by the doctor will also be low. In any such case, however, it is still probably true that, by implementing a dual procedure, when the patient had a life-expectancy beyond the upper limit, the doctor killed her patient. Because this probability was predictable *ex ante*, in many countries the dual procedure would at least amount to an *attempted* homicide, even if I am not aware of any physician ever to have been prosecuted on that score. It may be an attempted homicide, even when in actual fact the patient unexpectedly died after a few hours or days only.

That the dual procedure probably amounts to homicide in some form, when the life expectancy of the patient exceeds some upper limit, is a morally and legally relevant fact, even if any actual offence in this area will almost certainly fail to lead to prosecution and conviction.^38^*Prima facie* it justifies the prohibition of the dual procedure in this case, as it is stated by almost all guidelines. But by using the qualifier ‘prima facie’ I still want to leave it open whether any adequate justification can be provided for lifting the prohibition in some cases.

If both the patient’s underlying illness and his dehydrated condition are both necessary factors in the determination of the exact time of his death, we have a case of concurrent causation. One additional way in which systems of criminal law differ from each other concerns the way they deal with this phenomenon, and I cannot fully exclude the possibility that one or the other particular doctrine of concurrent causation might provide the doctor with a legal defense.^39^ To that extent my legal argument in this section can perhaps be defeated. But it is hard to see how such a legal oddity can correspond to a distinction with any moral significance. If you give a drug to a person, knowing that it has no lethal effects on any person of normal health, but has such effects on this particular person on account of a specific illness he is suffering from, surely you are fully responsible for the death of that person.

Are any other legal defenses open to the doctor in such cases? And if they are, do these options reflect morally significant facts? Three possibilities are worth considering.

1. In the Benelux-countries the law itself contains an exception to its prohibition of murder (Belgium), c.q. of taking someone’s life on his request (the Netherlands). However, the exception only applies to a doctor who has reported his action to a regional or national review committee, and has satisfied a number of substantial and procedural requirements of careful action. If the doctor has failed to report, in considering his case the public prosecution and eventually the court will still check his behaviour against these requirements. In the present case they will then normally have to conclude that the doctor has not asked an independent consultant to give his judgment of the case. More importantly, they will probably conclude that he has not used the medically appropriate means for ending a person’s life on his request. So, even if the fact that he ended the life of his patient will not necessarily be considered punishable by itself, the way in which he did it probably will be.^40^
2. In all countries the law already recognizes one exception to the prohibition of killing, to which only doctors can appeal. This concerns the hastening of death as a predictable side-effect of medical actions justified by their palliative aim, usually the use of morphine.^41^ The exception can be interpreted in two ways. In medical ethics and in some common law jurisdictions the doctor is supposed to be justified by the fact that he did not strictly intend the death of the patient, hut only took his death into account as a probable and proportionate side-effect.^42^ This justification is then usually considered a special application of a more general Doctrine of Double Effect. In other countries, including most European ones on the Calais side of the Channel, this is not a possible justification, because all that is required as the mental state component of criminal behaviour is foresight and endorsement. In these countries what matters instead is whether a medically recognized indication for action, e.g. the need to alleviate severe suffering, was available to the doctor, whether or not he actually acted on it. In German law, for example, he is then supposed to be justified by necessity, in other countries the ‘medical exception’ is a justificatory ground *sui generis*, whether it is explicitly recognized as such by a statute or court decision or not.

It is fairly common in the guidelines and in the literature to appeal to the medical exception, or rather to the Doctrine of Double Effect, in order to justify palliative sedation on its own, sometimes even when the authors deny that, if properly done, it can have any life-shortening effects at all.^43^ But sometimes the appeal is made for the more specific case of the dual procedure.^44^ If successful, the appeal might in particular show why, on the one hand, we need to stipulate a maximum life-expectancy, but, on the other, can permit ourselves to specify a relatively high maximum. For one essential element of the exception is a proportionality requirement: an unwanted side-effect is acceptable, but the cost should not be too high. Presumably that has been the argument for the two-weeks threshold in the Dutch and Flemish guidelines.

Whether this appeal succeeds, however, depends on the reasons for withholding artificial nutrition and hydration. That it is futile treatment, as these two guidelines suggest, is not an acceptable reason. In this case it is only the decision to sedate that is justified by its palliative aim, and dehydration is not an unavoidable side-effect of *that* decision. It is easily possible to prevent the occurrence of the ‘side-effect’ of the dual procedure as a whole: by administering fluids.^45^ That doesn’t endanger the palliative aim of the sedation. But if the shortening of the patient’s life is morally so problematic that you are prohibited to intend it, it cannot be ‘futile’ to administer fluids in order to prevent that effect.^46^ Hence the burden of proof is on the supporter of the appeal to the Doctrine to provide us with other reasons why we cannot consider artificial hydration. Only if he succeeds can he say: I intend to alleviate the patient’s suffering (by deep and continuous sedation), I intend to respect those other reasons, and I accept the shortening of the patient’s life as an unavoidable side-effect.

In § 2 I argued that the ‘salami-slicing’ techniques which I discussed in that section fail because by sedating a person one *disables* her to prevent the natural effects of the withdrawal of artificial hydration from occurring.^47^ One could object to that argument that in sedating the doctor didn’t have the *intention* to create that disabling effect. That would, according to this objection, be the crucial difference with the other cases I mentioned: incarcerating someone and failing to feed her, or dropping someone into the sea and allowing her to drown. In many countries this difference would, it is true, legally not be relevant, because the law is not interested in the intention of the doctor. But his intention could be morally relevant nevertheless, as the law seems to recognize in some other countries. That can only be the case, however, if the effect is unavoidable without giving up the justifying aim of the whole procedure. If you can avoid the effect, but don’t, and you have no stronger reasons than that it would be futile to try, you cannot protest that you didn’t intend that effect.

## The maximum life-expectancy

So if we consider the dual procedure of permanent sedation to full unconsciousness and withholding artificial nutrition and hydration, there is a certain threshold of life expectancy beyond which we cannot deny that the procedure probably amounts to a form of homicide The term ‘probably’ in this conclusion refers to a residual factual, not to any legal uncertainty.

What should the threshold be? As we have seen the professional guidelines give varying answers to this question, roughly in a range from 4 to 14 days.

Doctors’ predictions of the life expectancy of patients are known to be very unreliable beyond 3–4 days. Even a prediction of three days survival is pretty fallible; at the very least one should require the prediction to be confirmed by an independent expert. The most reliable sign for a short life-expectancy is the spontaneous reduction of the intake of fluids to less than circa 300 cc a day. Beyond 3–4 days, even a consensus judgment of several doctors has hardly any predictive value at all. For some forms of cancer the normal trajectory of the illness offers some footholds for a prognosis, but that prognosis will still not be accurate enough to differentiate between a life expectancy of 1, 2 or 3 weeks. In the case of other fatal illnesses estimations of life-expectancy are even less reliable.^48^ It is usually pointed out in this regard that doctors tend to overestimate rather than underestimate survival time.^49^ But that is only a statistical truth which leaves too much space for individual exceptions to be comfortable. In addition, doctors may have special biases in making their estimations in these cases, which should be of special concern.

Even if reliable estimations were possible, an upper limit of 14 days is certainly too high. As we have seen, it is based on the assumption that it normally takes about 2 weeks to die from dehydration. That is already at the upper end of the known range,^50^ but this is a range that applies to healthy people who start from a normal hydration status, not to patients who are in the final stage of a fatal illness.^51^ Patients in that category may be expected to die from dehydration after 3–4 days.

When artificial hydration is provided, estimations of life expectancy are unreliable beyond 3–4 days. When it is withdrawn and the patient does not die from his illness within 3–4 days, he may be expected to die from dehydration. Combining these data we appear to have reason to stipulate an upper limit of 3–4 days. (Remember that we cannot justify a few additional days by appealing to a proportionality requirement.) It seems even arguable that in all cases in which the patient has not spontaneously reduced the intake of fluids, by withholding artificial hydration from him after deep sedation we run a substantial risk of shortening his life.

Suppose a doctor, having sedated his patient and having withdrawn artificial hydration, is prosecuted for ending the life of his patient and it can, exceptionally, be proved that this patient has died from dehydration. If the patient had a life expectancy of more than 3–4 days, but less than 2 weeks, could the doctor appeal to some of the professional guidelines, in particular in countries in which guidelines, stipulating an upper limit of 2 weeks, have been adopted by a national professional organisation of doctors (the Netherlands and Flandres)?^52^ Although this will certainly help him, it should not exempt him, if the reasoning of those guidelines has itself been fallacious, for example by adopting any of the salami-slicing techniques which I discussed in § 2.

## Conclusion

In 1997 the USA Supreme Court denied the existence of a constitutionally guaranteed right to physician-assisted death in two landmark decisions.^53^ One of the arguments members of the Court used was that dying patients did not need to go through a period of intolerable suffering which could not be alleviated, because their doctor always had the option of sedation until death. It does not appear from the opinions of the judges that any of them realized that it is fairly common practice in the USA not to provide fluids to a dying patient who has been deeply sedated. As David Orentlicher pointed out in that same year, this means that in an unknown number of cases—actually probably a small number- the procedure the Court recommended, as it is actually practised, cannot be distinguished from the procedure it did permit to be forbidden.^54^

As we have seen, it will nearly always be impossible to establish in any concrete case with sufficient certainty that the dual procedure has actually hastened a patient’s death. That this is *ex ante* probable in all cases in which the life-expectancy of the patient exceeds a certain threshold, could have been a reason for the law to stipulate a specific prohibition, but this, as far as I know, has never been done by any statute or court decision. No doctor, as far as I know, has ever been convicted for killing on request, murder, manslaughter, attempted murder or any other crime against life for following the dual procedure when his sedated patient has died after, let’s say, 10 or 15 days. The law consistently leaves it to the profession to regulate this behaviour. And, indeed, many professional guidelines point out some threshold, but mostly in fairly vague terms, without explicating the reason for this requirement, and without ever making explicit how we should describe actions which do not respect it.

In most countries this means: euthanasia and physician-assisted suicide are legally forbidden, because of the sanctity of life, the vocation of the doctor to heal and not to kill, and/or because of the possibilities of abuse that allowing these actions is perceived to imply, but this particular form of killing by the doctor is left in a legal and moral limbo. All justifications of the prohibition of euthanasia and physician-assisted suicide are thereby compromised. If, for example, it is feared that the legalisation of euthanasia would lead to patients being killed because doctors or hospitals don’t expect sufficient remuneration for continued treatment, it is obvious that it will be much easier for them to achieve that aim by acting on a policy which does not require the consent of the patient in all cases, which is not monitored and controlled in any way, either by required consultation or by required reporting, and in which the causal nexus between the policy and the patient’s death is normally hard to prove. Similar considerations apply when the fear is that family-members coveting the inheritance will manipulate the patient and/or the doctor into arranging a physician-assisted death.

In the Netherlands, Belgium and Luxemburg the legal situation is almost equally unclear: in addition to the exception to the prohibition on killing someone on his request, explicitly stated in the law, another exception is not clearly foreclosed, even when it applies to cases which do not satisfy the legal requirements of due care. The reason for this cannot be that the rationale for these requirements does not fully apply to these cases.

As far as the guidelines are concerned, in all these countries clarity and consistency could be achieved by updating them in two ways:

1. The maximum life-expectancy for allowing the dual procedure should be reconsidered. As I have argued, a case can be made for stipulating that continuous deep sedation should only be considered when the patient has already spontaneously stopped eating and drinking. If it is preferred to stipulate a maximum life-expectancy of three or four days, expert confirmation by an independent consultant of the estimated life-expectancy should be required in all those cases in which the patient has not yet spontaneously stopped eating and drinking.
2. If this upper limit is foreseeably exceeded, it should be made explicit that the dual procedure in such a case will probably amount to some form of homicide, even if this will predictably be hard to prove. The relevant kind of homicide, whatever it is, will be one prohibited by the law, not covered by the usual ‘medical exception’. Only in the Benelux-countries it could conceivably be covered by a second exception to the prohibition of killing, but only if the relevant requirements of due care have been satisfied.

We have seen that the guidelines use several argumentative strategies in order to escape that conclusion: that we should consider the justifiability of continuous deep sedation and the withholding of hydration independently of each other, that the patient in any case has the legal right to refuse treatment, including artificial hydration, that the life-shortening effect is merely an unavoidable side-effect of a decision aimed at the alleviation of the patient’s severe suffering. I hope to have shown that all these arguments are fallacious, as well as inconsistent with the very requirement regarding the maximum life-expectancy itself. But the very fact that these arguments have been made so commonly shows that the conclusion that the dual procedure sometimes amounts to homicide is unwelcome.

It is easy to understand why. Cases occur in which it is an undeniable benefit for dying patients who have not already spontaneously stopped drinking, or have a life-expectancy beyond 3 or 4 days, to be deeply sedated until their death.^55^ But these same patients will often prefer not to have fluids administered to them, and this preference is also fully understandable. They may consider the uncertainties surrounding doctors’ assessments of the depth of a coma and the fact that the possibility of unwanted re-awakening cannot fully be excluded.^56^They may also consider how exhaustive it normally is for relatives (and medical staff) to wait for the end in such cases.

For these reasons an argument can be made that doctors should retain the option of choosing the dual procedure, up to a maximum life-expectancy of 2 weeks, or even beyond.^57^ This would require the law at least to explicitly create a second ‘medical exception’ to the prohibition of killing. But if the law is going to permit only one form of euthanasia, it is hard to see why it should be the slow one. The same reasons the patient may have for preferring continuous deep sedation without hydration he may cite for preferring euthanasia in its standard form. Which reasons can be given for not allowing it? The patient has nothing to gain, and both the patient and in particular his relatives have a lot to lose by this restriction. If the sanctity of life or the vocational integrity of the doctor is at stake in one practice, it is equally at stake in the other, and if the possibilities of abuse can be controlled in one case, as the guidelines obviously assume, they can equally be controlled in the other.

The appeal to a medical exception to the prohibition of killing which I considered in the last section presupposes that shortening the life of the patient, even in his actual state, is an unwelcome effect. The argument from futility I discussed in § 2 presupposes that it is at least an indifferent effect. But I have argued that if we permit the dual procedure in cases in which the life-expectancy of the patient exceeds three or four days, we actually imply that the effect is welcome. This assumption should be openly acknowledged.

If physician-assisted death in the particular form of the dual procedure is recognized as legal in some way, some regime of substantial and procedural requirements should be in place, perhaps similar to the regime of the Dutch and Belgian euthanasia laws. In these countries it should be made clear that these requirements apply to the dual procedure when the patient’s life-expectancy exceeds the upper limit. This will also mean that, in the case the patient survives longer than expected, it may still be an open option to use muscular relaxants in order to hasten his death. One important procedural requirement that is mostly not made at present is that the doctor who considers the dual procedure has to consult an independent palliative expert. These experts should give their informed opinion about the availability of alternative ways of alleviating the patient’s suffering, both pharmacological and non-pharmacological, about possible burdens these alternatives involve, and in particualr about the life expectancy of the patient.^58^

The dual procedure of starting continuous deep sedation and withholding hydration should be seen for what it is: a form of killing, in all cases in which the life-expectancy of the patient is beyond the maximum now recognized by the guidelines, but arguably also in some cases below that maximum, at least when it is put higher than at three or four days. Many guidelines at present stipulate a higher maximum, and all guidelines fail to clarify the moral and legal status of actions that do not respect the maximum. It is inconsistent at the same time to defend a general legal ban on euthanasia.

One person’s *Modus Ponens* is the other person’s *Modus Tollens*, and from the beginning the argument that the dual procedure at least sometimes amounts to euthanasia has been used in both ways.^59^ There are arguments on both sides, some of which I have mentioned. But though my position will be clear, it has not been my concern in this paper to argue for it.

## Footnotes

1 For criticism of that term see van Delden (2007), Battin (2008), Raus et al. (2012), den Hartogh (forthcoming).

2 Actually this is what many doctors understand ‘palliative sedation’ to mean, van Delden et al. (2011), p. 139.

3 Swart et al. (2012). The authors argue that a non-titrating policy can have legitimate aims; see also van Delden (2013), den Hartogh (forthcoming).

4 Maltoni et al. (2012), Sykes (2013), Barathi and Chandra (2013).

5 Morita et al. 2005, EAPC (2009). According to Lipuma (2013) deep sedation until death is itself a kind of euthanasia. In order to reach that conclusion he argues for a conception of death as the permanent, not necessarily irreversible loss of consciousness. If that conception would be the correct one, of two patients who are in exactly the same physiological condition one is dead and the other isn’t, depending on the intentions of their doctors.

6 Rady and Verheyde (2010) argue that deep sedation is likely to depress vital functions in the brainstem with possibly life-shortening effects. Cf. Leheup et al. (2012). If this is true, some appeal has to be made to a medical exception on the prohibition of killing, see below § 3.

7 According to Miccinesi et al (2006) it was decided to abstain from artificial nutrition and hydration in the following percentages of cases of palliative sedation: 35 % (Italy), 39 % (Flanders), 56 % (Sweden), 60 % (German Switzerland), 64 % (Denmark and the Netherlands). Later reports of similarly designed research concern the Netherlands 2005 (66 %, van Rietjens et al. 2008), Flanders 2007 (57 %, including 9 % withdrawal during sedation, Chambaere et al. 2010), and the Netherlands 2010 (79 %, van der Heide et al. 2012). We have no comparabale data for non-European countries, but for some indicative data see Bruinsma et al. (2013), p. 42.

8 The immediate cause of the patient’s death probably is heart failure, caused by a lack of kalium and natrium resulting from dehydration, V&VN/KNMG (2014), p. 25.

9 What do doctors think? According to Anquinet et al. (2011) 6 out of 28 physicians classified the policy as similar to euthanasia (2) or to ending of life without request (4). Van Delden et al (2011) asked doctors to evaluate a vignette-case in which the life-expectancy of the patient was a month, the patient was sedated and did not receive artificial hydration, and died after a week. Some respondents observed that this was ‘a kind of euthanasia’, but only few of them considered this a reason for a negative assessment of the decision.

10 Braun et al. (2003), Morita et al. (2005) (but elsewhere: days or weeks), HPNA (2008), EAPC (2009), Fraser Health (2011), NCCN (2013), section Palliative Sedation. Cf. Svenska Läkaresällskapets Delegation for Medicinsk Etik (2003), reported in: Førde et al. (2008): one week.

11 Quill and Byock (2000), De Graeff and Dean (2007), Dean et al. (2012).

12 NWA (2001), KNMG (2009), NHPCO (2010), Zorgnet Vlaanderen (2012). Cf. Morita et al. (2005): 2–3 weeks.

13 NEC (2006), NHPCO (2010).

14 Brody (1993), Craig (1994), Billings and Block (1996), Orentlicher (1997), Gauthier (2001), Battin (2008).

15 Sutcliffe and Holmes (1994), KNMG (2009), p. 36 with further references.

16 KNMG (2009), Zorgnet Vlaanderen (2012).

17 Known data about surviving time after sedation has been started (see footnote 38) seem to contradict it.

18 Quill and Byock (2000), Cowan and Palmer (2002), HPNA (2008), Morita et al. (2005), NEC (2006), De Graeff and Dean 2007, EAPC (2009), NHPCO (2010).

19 For extensive discussions of the benefits and burdens involved see De Graeff and Dean (2007), p. 76–77, Gurschick et al. (2014) As regards the balance of benefits and burdens it turns out that the available evidence does not allow us to draw any general conclusion. Cf. Good et al. (2008).

20 KNMG (2009), p. 37, is inconsistent on this point, first stressing that deeply sedating the patient ends her suffering, and then asserting that artificial hydration can prolong or exacerbate suffering, e.g. by causing edema or ascites. Let me add that for esthetic reasons it may be desirable to moisten the patient’s lips.

21 KNMG (2009), Neitzke et al. (2010), Zorgnet Vlaanderen (2012), NCCN (2013).

22 “One of the most difficult situations is where deep, permanent sedation is given to a patient who is expected to survive for more than one week. Some would argue that, in this situation, dehydration may hasten death. Others would argue that giving fluids would neither prevent death, nor make it more comfortable, but merely prolong the dying process. There is no evidence to support either view.” De Graeff and Dean (2007), p. 76. But as regards their empirical content, both views are correct, and fully compatible.

23 The ‘neutral container’ view of the value of life, Kagan (2012), 258ff, cf. Raz (2001), ch. 3.

24 This has most prominently been defended by Germain Grisez, Joseph Boyle and John Finnis in many works, e.g. Finnis et al. (1987). In their view biological life even is a fundamental good that cannot be out-balanced by any other fundamental goods.

25 Cf. IACB (2012), referring to Congregation for the Doctrine of the Faith (2007).

26 De Graeff and Dean (2007), NEC (2006), EAPC (2009). Cf. the debate about ending artificial feeding of patients in a persistent vegetative state, as in the famous case *Airedale NHS Trust v. Bland* (1993) AC 789 HL. (Or, rather, an Unresponsive Wakefulness Syndrome, cf. von Wildt et al. 2012).

27 The argument has most explicitly been stated by Tännsjö (2004a). It is implied by all authors who suggest that the decision to abstain from providing artificial fluids should be judged in the same way as other decisions to withhold or withdraw treatment, e.g. Williams (2001), Lewis (2008), p. 359, Gevers (2002), p. 365, Delbeke (2013), p. 144. Guidelines taking that position include AMA (2008), EAPC (2009), and the guidelines mentioned in footnote 21.

28 That counter-example came from Kuhse (2004), p. 61: Frieda putting her kitten into a box and deliberately leaving it there without food and water until it dies.

29 For criticism see den Hartogh (2014).

30 Orentlicher (1997), Kuhse (2004), Van Delden (2013), 218–227.

31 Delbeke (2013).

32 Orentlicher (1997), Hallenbeck (2000), Cowan and Palmer (2002), Cantor (2006), Quill et al. (2009), Delbeke (2013), Holm (2013), p. 232. Note again that, if the argument would be valid, it would justify the dual procedure, irrespective of the life-expectancy of the patient.

33 As for the patient, if she has consented to the sedation and refused to consent to artificial hydration, she has by implication consented to the dual procedure.

34 Van Delden (2013), p. 219.

35 Suppose that a patient decides to end her life by stopping eating and drinking, and as part of this plan refuses artificial nutrition and hydration, regardless of whether or not she will be sedated. In that case her refusal is an independent decision. If during that process a doctor decides to start deep sedation, could this amount to homicide? My answer is: yes, when by sedating the patient he disables her to change her mind. But suppose that the patient gets into a delirium when she starts losing consciousness. If we should consider stopping eating and drinking a form of suïcide (cf. footnote 29), the sedation should, I suggest, then be seen as a kind of assistance, which, in legal systems with a general prohibition of assisting suicide, might require a special legal defense.

36 Horder (2007).

37 For example, *dolus eventualis* (death as a result is merely possible, but the possibility is ‘willingly embraced’) is enough for murder in Germany and the Netherlands, but not in France or the UK.

38 The following data about surviving time have been reported: the Netherlands 2005: 4 % survival of 1–2 weeks, 2 % of more than 2 weeks (van Rietjens et al. 2008), Belgium 2007: 6 % 1–2 weeks, 3 % more than 2 weeks (Chambaere et al. 2010), UK 2007: 8,3 % more than 1 week (Seale 2010), the Netherlands 2010: 2 % 1–2 weeks, 1 % more than 1 week (Van der Heide et al. 2010. The drop in the Netherlands between 2005 and 2010 can be ascribed to the publication of the KNMG guideline in 2005. Unfortunately, these figures don’t differentiate between cases in which artificial hydration has and has not been provided.

39 Cf. footnote 52 below.

40 The Dutch guideline has been developed in 2005 by the Royal Dutch Society of Medicine (KNMG) in response to a paper of the Director of Public Prosecutions arguing that the dual procedure might be a kind of homicide, de Wijckerslooth (2003). After the guideline had been published, the Director announced (too quickly to my mind, see § 4) that no doctor who has complied with the guideline will be prosecuted, Griffiths et al. (2008 p. 61). Unfortunately the legal status of actions not satisfying the requirement of the maximum life-exectancy has been left undefined since then.

41 Even though the present consensus is that the medically appropriate use of morphine for palliative reasons has no life-shortening effects either. As I have stated it, the exception is a special case of a wider medical exception, that is certainly needed to justify continuous deep sedation. Otherwise it would be a form of battery.

42 Actually the legal position is often either more complex or less clear or both than this formulation suggests. English law in particular is both opaque and ambiguous, see Price (1997), Ashworth and Holder (2009), p. 238, Williams (2007), ch. 1, Lewis, p. 350.

43 E.g. Cowan and Palmer (2002), HPNA (2008), Lo and Rubenfeld (2005), De Graeff and Dean (2007), 76–77, AMA (2008), Boyle (2004), Rousseau (2004), Levy and Cohen (2005), Carr and Mohr (2008), Baumann et al. (2011), Leheup et al. (2012).

44 Sulmasy and Pellegrino (1999), NCCN (2013), De Graeff and Dean (2007), den Hartogh (2006), McIntyre (2014), Van Delden (2013).

45 Orentlicher (1997), G. Williams (2001), Raus et al. (2013, p. 199), Holm (2013).

46 Holm (2013).

47 Or by appealing to some principle of double effect, see below. See on the authority of the guidelines Delbeke (2013), at p. 135, cf. footnote 40.

48 See Van Delden (2013) for a more detailed analysis.

49 Glare et al. (2003). The overestimation is on average 30 %, and the larger the longer the expected time of survival.

50 Chabot (2008), p. 28.

51 Van Delden (2013), p. 221.

52 A Dutch doctor, for example, could argue, as follows. He could concede that the dual procedure he used had a life-shortening effect, but insist that the patient’s underlying illness has also been a necessary factor in the determination of the moment of death. He could then argue that, of these two concurrent causes, the death of the patient could only be ‘reasonably attributed’ to the patient’s illness, because his own actions had fully met the requirements of the KNMG-guideline. Cf. Rozemond (2009).

53 *Washington v Glucksberg*, 117 S. Ct. 22558 (1997), opinions of Justices O’Connor and Breyer, joined by Justice Ginsburg. The argument that sedation until unconcsiousness is ‘covert assisted suicide’ is rejected in *Vacco v. Quill* S Ct. 2293 (1997).

54 Orentlicher (1997).

55 Either because they are suffering from refractory symptoms -the only reason recognized by the guidelines- or for other reasons, see den Hartogh (forthcoming).

56 Noreika et al. (2011), DeSchepper et al. (2013). Morita (2005), Anquinet et al. (2011) (8 out of 28 patients woke up due to insufficient medication).

57 Cf. Cellarius (2008).

58 At present most guidelines recommend but do not require this, except for cases in which existential suffering is the primary reason for sedation, De Graeff and Dean (2007), Dean et al. (2012). Consultation is ‘essential’ according to Braun et al. (2003), NEC (2006), AMA (2008), HPNA (2008).

59 Used as an argument for the legalisation of physician-assisted death, this is an appeal to consistency of a type, normally exemplified in the literature by throwing doubt on the moral relevance of the distinctions between killing and letting die, and between causing death as an intended and as a merely foreseen effect, see e.g. Sumner (2011). But the cogency of this particular argument does not depend on the success of these structurally similar ones.

## References

[CR1] AMA (American Medical Association, Council on Ethical and Judicial Affairs). 2008 *Sedation to Unconsciousness in End*-*of*-*life Care.* CEJA Report 5-A-08.

[CR2] Anquinet L, Rietjens JAC, van den Block L, Bossuyt N, Deliëns L (2011). General practitioners’ report of continuous deep sedation until death for patients dying at home: A descriptive study from Belgium. European Journal of General Practice.

[CR3] Ashworth A, Horder J (2009). Principles of criminal law.

[CR4] Barathi B, Chandra PS (2013). Palliative sedation in advanced cancer patients: does it shorten life? A systematic review. Indian Journal of Palliative Care.

[CR5] Battin MP (2008). Terminal sedation: Pulling the sheet over our eyes. Hastings Center Report.

[CR6] Baumann, A., F. Claudot, G. Audibert, and P-M Mertes, L. 2011. Puybasset, Sedation in severely brain-injured patients: A French perspective. *Philosophy, Ethics and Humanities in Medicine* 6 (4). 10.1186/1747-5341-6-4 PMC3041748 21303504

[CR7] Billings JA, Block SD (1996). Slow euthanasia. Journal of Palliative Care.

[CR8] Boyle J (2004). Medical ethics and double effect: The case of terminal sedation. Theoretical Medicine and Bioethics.

[CR9] Braun TC, Hagen NA, Clark T (2003). Development of a clinical practice guideline for palliative sedation. Journal of Palliative Medicine.

[CR10] Brody H (1993). Causing, intending and assisting death. Journal of Clinical Ethics.

[CR11] Bruinsma, S.M., J.A.C. Rietjens and A. van der Heide. 2013. Continuous sedation until death: State of the Art. In: Sterckx, Raus and Mortier eds. 2013: 29–46.

[CR12] Cantor NL (2006). On hastening death without violating legal and moral prohibitions. Loyola University Chicago Law Journal.

[CR13] Carr MF, Mohr GJ (2008). Palliative sedation as part of a continuum of palliative care. Journal of Palliative Medi- cine.

[CR14] Cellarius V (2008). Terminal sedation and the ‘imminence condition’. Journal of Medical Ethics.

[CR15] Chabot, B. 2008. *A hastened death by self*-*denial of food an drink*. Amsterdam.

[CR16] Chambaere K, Bilsen J, Cohen J, Rietjens JA, Onwuteaka-Philipsen BD, Mortier F, Deliëns L (2010). Continuous deep sedation until death in Belgium: A nationwide survey. Archives of Internal Medicine.

[CR17] Congregation for the Doctrine of the Faith. 2007. Responses to certain questions of the United States Conbference of Catholic Bishops concerning Artificial Nutrition and Hydration.

[CR18] Cowan JD, Palmer TW (2002). Practical guide to palliative sedation. Current Oncology Reports.

[CR19] Craig G (1994). On witthholding nutrition and hydration in the terminally ill: has palliative medcine gone too far?. Journal of Medical Ethics.

[CR20] Dean MM, Cellarius V, Henry B, Oneschuk D, Librach SL, (Canadian Society Of Palliative Care Physicians Taskforce) (2012). Framework for continuous palliative sedation therapy in Canada. Journal of Palliative Medicine.

[CR34] De Graeff A, Dean M (2007). Palliative sedation therapy in the last weeks of life: A literature review and recommendations for standards. Journal of Palliative Medicine.

[CR21] Delbeke, E. (2013) The legal permissiblity of continuous deep sedation at the end of life: a comparison of laws and a proposal, in: Sterckx, Raus and Mortier eds.: 132–148.

[CR100] den Hartogh, G.A. 2006. Palliatieve sedatie en euthanasie: Commentaar op een richtlijn. Tijdschrift voor Gezondheidsrecht 30: 109–119.

[CR101] den Hartogh, G.A. 2014. De rol van de arts bij levensbeëindiging door stoppen met eten en drinken. Commentaar op de concepthandreiking van de KNMG. Tijdschrift voor Gezondheidsrecht 38: 192–200.

[CR102] den Hartogh, G.A. forthcoming. Sedation until death: are the requirements laid down in the guidelines too restrictive? Kennnedy Institute of Ethics Journal. 10.1353/ken.2016.0035 28533496

[CR25] Deschepper R, Laureys S, Hachimi-Idrissi S, Poelaert J, Bilsen J (2013). Palliative sedation: why we should be more concerned that patients experience an uncomfortable death. Pain.

[CR26] EAPC (European Association for Palliative Care) (2009). Recommended framework for the use of sedation in palliative care. Journal of Palliative Medicine.

[CR27] Finnis J, Boyle J, Grisez G (1987). Nuclear deterrence, morality, and realism.

[CR28] Førde R, L.J. Materstvet, and A. Syse. 2011. Scandinavia, in: J. Griffiths, H. Weyers, M. Adams, eds, *Euthanasia and Law in Europe*. Oxford: Hart, 2008: 425–441.

[CR29] Fraser Health. 2011. Refractory Symptoms and Palliative Sedation: Therapy Guideline.

[CR30] Gauthier CC (2001). Active voluntary euthanasia, terminal sedation and assisted suicide. Journal of Clinical Ethics.

[CR31] Gevers S (2002). Terminal sedation: A legal approach. European Journal of Health Law.

[CR32] Glare P, Virik K, Jones M, Hudson M, Eychmuller S, Symes J, Chirstakis N (2003). A systematic review of physicians’ survival predictions in terminally ill cancer patients. British Medical Journal.

[CR33] Good, P., J. Cavenagh, M. Mather, and P. Ravenscroft. 2008. Medically assisted hydration for palliative care patients. *Cochrane Database Syst Rev* (2). CD006273. 10.1002/14651858.CD006273.pub2 18425944

[CR35] Griffiths J, Weyers H, Adams M (2008). Euthanasia and law in Europe.

[CR36] Gurschick, L, D.K. Mayer and L.C. Hanson, 2014. Palliative sedation: An analysis of international guidelines and position statements. *American Journal of Hospice and Palliative Medicine*, publ. online 7 May. 10.1177/1049909114533002 24807825

[CR37] Hallenbeck JL (2000). Terminal sedation: Ethical implications in different situations. Journal of Palliative Medicine.

[CR39] Holm, S. 2013. Terminal sedation and euthanasia: the virtue in calling a spade what it is. In: Sterckx, Raus and Mortier eds.: 221–239.

[CR40] Horder J (2007). Homicide law in comparative perspective.

[CR41] HPNA (Hospice and Palliative Nurses Association) (2008). HPNA Position Statement: Palliative Sedation.

[CR42] IACB (International Association of Catholic Bioethicists). 2012. The Use of Sedatives in the Care of Persons who are Seriously Ill or Dying: Ethical Distinctions and Practical Recommendations. *National Catholic Bioethics Quarterly* 12: 494–501. Quoted from *Bioethics Outlook* (Plunkett Centre for Ethics) 24 (2013) no 2: 1–12.

[CR43] Kagan S (2012). Death.

[CR44] KNMG (Koninklijke Nederlandsche Maatschappij tot Bevordering der Geneeskunst) 2009. *Guideline Palliative Sedation*, revised edition. (First edition 2005).

[CR45] Kuhse, H. 2004. Why Terminal Sedation is No Solution to the Voluntary Euthanasia Debate, in: Tännsjö ed. 2004.

[CR46] Leheup BFE, Piot X, Ducrocq B Wary (2012). Théorie du double effet et sédation pour détresse en phase terminale: réflexion autor de la survie des patients sédatés. Presse Medicale.

[CR47] Levy MH, Cohen SD (2005). Sedation for the relief of refractory symptoms in the imminently dying: a fine intentional line. Seminars in Oncology.

[CR48] Lewis, P. 2008. England and Wales, in: Griffiths et al. eds.

[CR49] Lipuma SH (2013). Continuous deep sedation until death as physician-assisted suicide.euthanasia: a conceptual analysis. Journal of Medicine and Philosophy.

[CR50] Lo B, Rubenfeld G (2005). Palliative sedation in dying patients: ‘We turn to it when everything else hasn’t worked’. JAMA.

[CR51] Maltoni M, Scarpi E, Rosati M, Derni S, Fabbri L, Martini F, Amadori D, Nanni O (2012). Palliative sedation in end-of-life care and survival: a systematic review. Journal of Clinical Oncology.

[CR52] McIntyre, A. 2014. Doctrine of Double Effect, *Stanford Encyclopedia of Philosophy*.

[CR53] Miccinesi G, Rietjens JAC, Deliëns L, Paci E, Bosshard G, Nilstun T, Norup M, van der Wal G (2006). Continuous deep sedation: Physicians’ experiences in six European countries. Journal of Pain and Symptom Management.

[CR54] Morita T, Bito S, Kurihara Y, Uchitomi Y (2005). Development of a clinical guideline for palliative sedation therapy using the delphi method. Journal of Palliative Medicine.

[CR55] Morita T, Chinone Y, Ikenaga M (2005). Efficacy and safety of palliative sedation therapy: a multicenter, prospective, observational study conducted on specialized palliative care units in Japan. Journal of Pain and Symptom Management.

[CR56] NCCN (National Comprehensive Cancer Network). 2013. *Clinical practice guidelines in oncology: palliative care.* Updated 2013. 10.6004/jnccn.2009.0031 19406043

[CR57] NEC (National Ethics Committee of the Veterans Health Administration). 2006. *The Ethics of Palliative Sedation: A Report.*

[CR58] Neitzke G, Oehmichen F, Schliep HJ, Wördehoff D (2010). Sedierung am Lebensende: Empfehlungen der AG Ethik am Lebensende in der Akademie für Ethik in der Medizin (AEM). Ethik in der Medizin.

[CR59] NHPCO (National Hospice and Palliative Care Organization) (2010). Position statement and commentary on the use of palliative sedation in imminently dying terminally ill patients. Journal of Pain and Symptom Management.

[CR60] Noreika V, Jylhuankangas L, Móró L, Valli K, Kaskinoro K, Aantas R, Scheinin H, Revonsuo A (2011). Consciousness lost and found: subjective experiences in an unresponsive state. Brain and Cognition.

[CR61] NWA (Norwegian Medical Association), Medical Ethics Council, 2001. Guidelines for palliative sedation of the dying. In: Tännsjö ed. 2004, Appendix, p. 132–133.

[CR62] Orentlicher D (1997). The Supreme Court and terminal sedation: rejecting assited suicide, embracing euthanasia. Hastings Constitutional Law Quarterly.

[CR63] Price D (1997). Euthanasia. Pain Relief and Double Effect, Legal Studies.

[CR64] Quill TE, Byock IR, (American College of Physicians/American Society of Internal Medicine) (2000). Responding to intractable terminal suffering: the role of terminal sedation and voluntary refusal of food and fluids. Annals of Internal Medicine.

[CR65] Quill TE, Lo B, Brock DW, Meisel A (2009). Last-Resort options for palliative sedation. Annals of Internal Medicine.

[CR66] Rady MY, Verheijde JL (2010). Continuous deep sedation until death: palliation or physician-assisted death?. American Journal of Hospice and Palliative Medicine.

[CR67] Raus K, Sterckx S, Mortier F (2012). Continuous deep sedation at the end of life and the ‘natural death’ hypothesis. Bioethics.

[CR68] Raus, K, S. Sterckx, and F. Mortier. 2013. Can the doctrine of double effect justify continuous deep sedation at the end of life? In: Sterckx, Raus and Mortier eds., 2013: 177–2001.

[CR69] Raz J (2001). Value, respect, and attachment.

[CR70] Rietjens JA, van Delden J, Onwuteaka-Philipsen B, Buiting H, van der Maas P, van der Heide A (2008). Continuous deep sedation for patients nearting death in the Netherlands: descriptive study. British Medical Journal.

[CR71] Rousseau P (2004). Palliative sedation in the management of refractory symptoms. Journal of Supportie Oncology.

[CR72] Rozemond K (2009). Palliatieve zorg of moord: causaliteit is de crux. Tijdschrift voor Gezondheidsrecht.

[CR73] Seale C (2010). Continuous deep sedation in medical practice: A descriptive study. Journal of Pain and Symptom Management.

[CR74] Sterckx S, Raus K, Mortier F (2013). Continuous sedation at the end of life: Ethical.

[CR75] Sulmasy DP, Pellegrino ED (1999). The rule of double effect: clearing up the double talk. Archives Internal Medicine.

[CR76] Sumner LW (2011). Assisted death: a study in ethics and law.

[CR77] Sutcliffe J, Holmes S (1994). Dehydration: Burden or benefit to the dying patient?. Journal of Advanced Nursing.

[CR78] Swart SJ, van der Heide A, van Zuylen L, Perez RSGM, Zuurmond WWA, van der Maas PJ, van Delden JJM, Rietjens JAC (2012). Considerations of physicians about the depth of palliative sedation at the end of life. Canadian Medical Association Journal.

[CR79] Sykes, N.P. 2013.* Clinical Aspects of palliative sedation*. In: Sterckx, Raus and Mortier eds. 2013: 86–99.

[CR80] Tännsjö, T. 2004a. Terminal sedation: A substitute for Euthanasia? In: T. Tännsjö ed.

[CR81] Tännsjö T (2004). Terminal sedation: euthanasia in disguise?.

[CR22] Van Delden JJM (2007). Terminal sedation: Source of a restless ethical debate. Journal of Medical Ethics.

[CR23] Van Delden, and J.J.M. 2013. The ethical evaluation of continuous sedation at the end of life. In: Sterckx, Raus and Mortier eds.: 218–227.

[CR24] Van Delden JJM, van der Heide A, van de Vathorst S, Weyers H, van Tol DG (2011). Kennis en opvattingen van publiek en professionals over medische besluitvorming en behandeling rond het einde van het leven: Het KOPPEL-onderzoek.

[CR38] Van der Heide A, Brinkman-Stoppelenburg A, van Delden H, Onwuteaka-Philipsen B (2012). Euthanasie en andere medische beslissingen rond het levenseinde: Sterfgevallenonderzoek 2010.

[CR82] Von Wild K, Laureys ST, Gerstenbrand F, Dolce G, Onose G (2012). The vegetative state—A syndrome in search of a name. Journal of Medicine and Life.

[CR83] V&VN/KNMG. 2014. *Zorg voor mensen die bewust afzien van eten en drinken om het levenseinde te bespoedigen: een handreiking*. Utrecht.

[CR84] Williams G (2001). The principle of double effect and terminal sedation. Medical Law Review.

[CR85] Williams G (2007). Intention and causation in medical non-killing: The impact of criminal law concepts on euthanasia and assisted suicide.

[CR86] Wijckerslooth, J.I. de. 2003. Twee lacunes in de euthanasieregeling, *Opportuun.* nr 10.

[CR87] Zorgnet Vlaanderen, 2012. *Richtlijn Sedatie.*

